# Co-Application of Eugenol and QX-314 Elicits the Prolonged Blockade of Voltage-Gated Sodium Channels in Nociceptive Trigeminal Ganglion Neurons

**DOI:** 10.3390/biom10111513

**Published:** 2020-11-05

**Authors:** Sung-Min Hwang, Kihwan Lee, Sang-Taek Im, Eun Jin Go, Yong Ho Kim, Chul-Kyu Park

**Affiliations:** 1 Gachon Pain Center and Department of Physiology, Gachon University College of Medicine, Incheon 21999, Korea; unclehwang76@gmail.com (S.-M.H.); key1479@gmail.com (K.L.); sangtaek.im57@gmail.com (S.-T.I.); navy-2474@hotmail.com (E.J.G.); 2 Fight against Angiogenesis-Related Blindness (FARB) Laboratory, Seoul National University Hospital, Seoul 03082, Korea

**Keywords:** local anesthetic, eugenol, QX-314, voltage-gated sodium channels, TRPV1, nociception, trigeminal ganglion

## Abstract

Local anesthetics (LAs) can completely block nociception by inhibiting voltage-gated sodium channels (VGSCs), and thus, blocking action potentials (APs) within sensory neurons. As one of the several LAs, eugenol is used for dental pain treatment. It reportedly features multiple functions in regulating diverse ion channels. This study aimed to investigate the long-lasting analgesic effect of eugenol alone, as well as that of the combination of eugenol as a noxious-heat-sensitive transient receptor potential vanilloid 1 (TRPV1) channel agonist and a permanently charged sodium channel blocker (QX-314), on neuronal excitability in trigeminal ganglion (TG) neurons. Eugenol alone increased inward current in a dose-dependent manner in capsaicin-sensitive TG neurons. Eugenol also inhibited the VGSC current and AP. These effects were reversed through wash-out. The combination of eugenol and QX-314 was evaluated in the same manner. The combination completely inhibited the VGSC current and AP. However, these effects were not reversed and were continuously blocked even after wash-out. Taken together, our results suggest that, in contrast to the effect of eugenol alone, the combination of eugenol and QX-314 irreversibly and selectively blocked VGSCs in TG neurons expressing TRPV1.

## 1. Introduction

Recent studies on new local anesthetics (LAs) have focused on several major goals to separate sensory and motor nerve block, lengthen the duration of analgesic action, and reduce the number of side effects [1,2]. Lidocaine, one of the best known LAs, achieves a local aesthetic effect by blocking voltage-gated sodium channels (VGSCs) [3,4]. However, lidocaine action is non-selective and blocks action potentials (APs) in all sensory motor and autonomic fibers [3,4,5]. Lidocaine is limited by its relatively short duration of action, which is usually insufficient to completely cover the normal duration of post-operative pain management [6]. Furthermore, major central nervous system and cardiovascular toxicities may occur when it is administered locally at high volumes due to the effects of lidocaine on neuronal cells and cardiac muscles [7]. Therefore, a pharmacological therapy with a high selectivity for nociceptors, a longer duration of analgesia, and a reduced burden of adverse effects is needed.

One analgesic approach is to block only specific subtypes of sensory neurons using QX-314; a positively charged derivative of lidocaine with a molecular mass of 263 Da. QX-314 cannot pass through the plasma membrane unaided and blocks neuronal sodium channels only when applied intracellularly [3,7,8]. A number of nonselective cation channels can permit the entry of positively charged large molecules (600 Da) through their conduction pores, thereby enabling the selective intracellular access of specific molecules to the cells expressing these channels [3]. The transient receptor potential vanilloid 1 (TRPV1) channel is a noxious heat detector with a pore that is large enough to allow for the permeation of cationic drugs such as QX-314 [3]. Therefore, the co-administration of capsaicin and QX-314 prompts the influx of QX-314 into the neurons through TRPV1. This combined pharmacotherapy has been reported to dramatically inhibit the sodium current and abolish the ability of TRPV1-expressing dorsal root ganglion neurons to generate APs [9,10]. The inhibitory effect on the sodium current and the AP in TRPV1-expressing trigeminal ganglion (TG) neurons was irreversible after wash-out of capsaicin and QX-314 [9,10]. In contrast to lidocaine, the irreversible condition and the long duration of the sodium current blocking effected by QX-314 is probably due to its inability of diffusing out of the membrane and its consequently being trapped within the axon [11]. However, under these specific conditions, TRPV1 activation by capsaicin occurs immediately (<1 s), while the entry of enough QX-314 to block APs takes several minutes [11]. This delay is long enough for capsaicin administration to enable a high level of nociceptive receptor activation for several minutes, which can cause a severe burning pain in humans [11]. Hence, using nonpungent agonists of TRPV1 other than capsaicin is preferred to overcome painful irritation through TRPV1 activation in local tissue.

Eugenol has been primarily used as a local analgesic drug in dentistry practice and produces an anesthetic effect through VGSC inhibition in the dental primary afferent neurons of rats [12,13]. Eugenol also modulates various ion channels that are responsible for nociception and neuronal activity, leading to its analgesic effects [13,14,15,16,17]. However, local analgesic actions, caused by eugenol, are generally relatively short-lived and induce a non-selective blockade, which is inadequate for the treatment of prolonged acute and chronic pain [10,18]. In such instances, achieving prolonged sensory nerve and selective pain blockade is desired. In addition to the analgesic effects of eugenol alone, the possibility of prolonged sensory nerve and selective pain blockade is achieved through eugenol-induced TRPV1 activity and QX-314 [10,12,13,17,18,19]. This study aimed to compare the reversibility of eugenol alone with that of the combination of eugenol and QX-314 on neuronal excitability in TG neurons.

## 2. Materials and Methods

### 2.1. Animals

All surgical and experimental procedures were reviewed and approved by the Institutional Animal Care and Use Committee of the College of Medicine at Gachon University (approval number: LCDI-2018-0064 01 June 2018). Animal treatments were performed according to the guidelines of the International Association for the Study of Pain. Adult male Sprague—Dawley rats were purchased from OrientBio Inc. (Sungnam, Korea). Animals were acclimatized in a conventional facility with a 12:12 h light—dark cycle (lights were turned on at 8:00 am) for at least 1 week prior to experiments, and they had ad libitum access to water and food.

### 2.2. Preparation of TG Neurons

The TG neurons from adult Sprague–Dawley rats were prepared as previously described [2,13,20]. Briefly, TG neurons were maintained in Hank’s balanced salt solution (HBSS; Welgene, Daegu, Korea) at 4 °C and then incubated at 37 °C for 60 min in 2 mL of HBSS containing 0.25% trypsin (Invitrogen, Carlsbad, CA, USA). The cells were washed, triturated with a fire-polished Pasteur pipette, and placed on 0.5 mg/mL poly-l-ornithine-coated glass coverslips (Sigma, St. Louis, MO, USA). The cells were maintained in a 5% CO_2_ incubator at 37 °C, and they were used for patch-clamp recording within 8 h after being plated.

### 2.3. Whole-Cell Patch-Clamp Recordings

The whole-cell configuration of the patch-clamp technique was performed with an Axopatch-200B amplifier (Axon Instruments, Union City, CA, USA). The resistance of patch electrodes was 4-6 MΩ. The recording chamber (volume 300 μL) was continuously superfused (2–3 mL/min. The pipette solution was composed of the following (in mM): 135 CsCl, 30 CsOH, 2 Mg-ATP, 10 HEPES, and 5 EGTA; its pH was adjusted to 7.3 with NaOH. The bath was continuously perfused with extracellular solution, which was composed of the following (in mM): 140 NaCl, 5 KCl, 1 MgCl_2_, 10 HEPES, 10 glucose, and 2 EGTA; pH of the latter was adjusted to 7.3 with NaOH. Partial series resistance compensation was used, and currents were low-pass filtered at 2 kHz and sampled at 10 kHz. P-Clamp10 (Axon Instruments) software was used during the experiments and analysis. The pipette solution for current-clamp experiments contained the following (in mM): 145 K-gluconate, 2 MgCl_2_, 1 CaCl_2_, 10 EGTA, 5 HEPES, and 5 K_2_ATP; its pH was adjusted to 7.3–7.4 with KOH. The extracellular solution for current-clamp experiments contained the following (in mM): 140 NaCl, 5 KCl, 2 CaCl_2_, 1 MgCl_2_, 10 HEPES, and 10 glucose; its pH was adjusted to 7.4 with NaOH. Electrophysiological recordings were used only one neuron in a coverslip, including a 1–2 min baseline, 10 min eugenol treatment, and 5 min wash-out, 1–2 min eugenol treatment, and 5 min wash-out session. After the eugenol treatment, 1μM capsaicin applied at the end to check the recording status.

### 2.4. Drugs

Capsaicin and eugenol were purchased from Sigma (St. Louis, MO, USA). QX-314 (*N*-(2,6-dimethylphenyl carbamoylmethyl) triethylammonium chloride) was obtained from Tocris (Bristol, UK). Eugenol and capsaicin were dissolved in dimethylsulfoxide (DMSO) or ethanol to make stock solutions and kept at −20 °C. The final concentration of DMSO was less than 0.1% (*v*/*v*), which did not affect the membrane currents. QX-314 was dissolved in distilled water. The drugs were diluted to their final concentration with the extracellular solution. The perfusion system was driven by gravity and a flow speed of 3–4 mL/min.

### 2.5. Statistical Analysis

All data were expressed as means ± standard errors of the means. One-way analysis of variance (ANOVA) or unpaired Student’s t-test was used to determine statistically significant differences. These tests were performed using Origin 6.0 (Microcal Software Inc., MA, USA) and Prism 7.0 (GraphPad Software, CA, USA). *p* Values < 0.05 were considered statistically significant.

## 3. Results

### 3.1. Eugenol Inhibited VGSC Currents in Small-Sized TG Neurons

**Figure 1:**
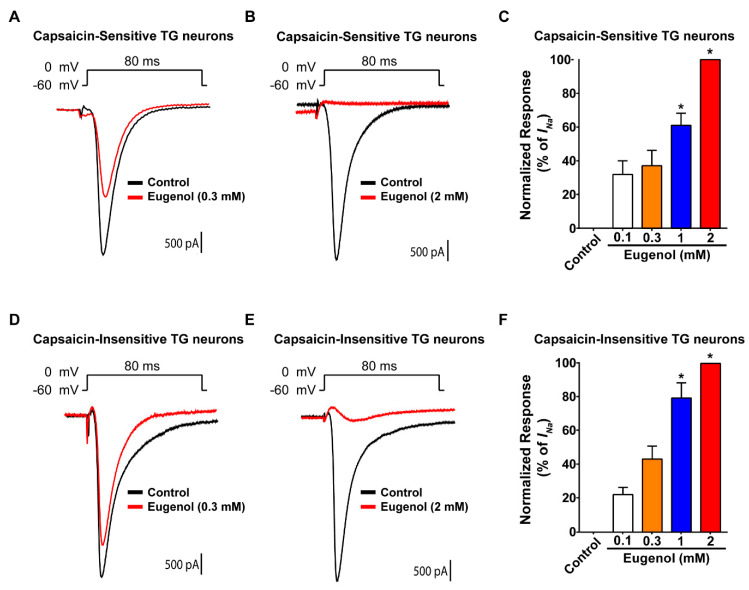
Effects of eugenol on voltage-gated sodium channel currents in small-sized TG neurons. Long-time base recordings of voltage-gated sodium channel (VGSC) currents measured during 80 ms-voltage steps from a holding potential of −60 mV to a potential of 0 mV delivered every 3 s. (**A**) Representative VGSC current recording following the application of 0.3 mM eugenol and (**B**) 2 mM eugenol. (**C**) Percentage inhibition of VGSC currents by eugenol in capsaicin-sensitive TG neurons (from 0.1 to 2 mM, *n* = 37). (**D**) Representative VGSC current recording following the application of 0.3 mM eugenol and (**E**) 2 mM eugenol. (**F**) Proportion of VGSC current inhibition effected by eugenol (from 0.1 to 2 mM, *n* = 29) in capsaicin-insensitive TG neurons. * *p* < 0.05 versus control (no treatment).

VGSC currents are responsible for the initial depolarization phase involved in AP generation, which results in pain signaling [21]. Thus, we evaluated the effect of eugenol on VGSC currents in cultured small-sized TG neurons using a command pulse from a holding potential stepped from −60 to 0 mV. At the end of each recording, capsaicin-sensitive and -insensitive TG neurons were identified by the presence or absence of an inward current caused by 1μM capsaicin (data not shown). In capsaicin-sensitive TG neurons, 0.3 mM of eugenol slightly decreased VGSC currents relative to control levels (Figure 1A), whereas 2 mM of eugenol completely inhibited VGSC currents (Figure 1B). Additionally, in capsaicin-insensitive TG neurons, 0.3 mM of eugenol partially reduced VGSC currents (Figure 1D), whereas 2 mM of eugenol completely blocked VGSC currents (Figure 1E). These results demonstrated that 2 mM of eugenol completely inhibited VGSC regardless of the presence of the capsaicin response in TG neurons (Figure 1C,F).

### 3.2. Eugenol Inhibited Aps in Small-Sized TG Neurons

**Figure 2:**
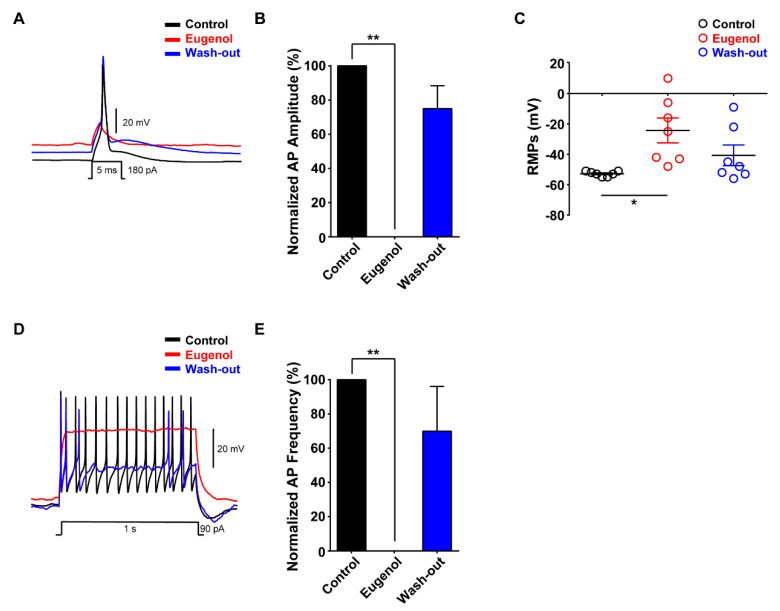
Effect of eugenol on action potentials in small-sized TG neurons. (**A**) Eugenol (2 mM) inhibited action potentials (APs) following current injection (5 ms, 180 pA) in TG neurons. Traces of single APs were observed before (control) and after treatment with eugenol. (**B**) All data were normalized to controls (black bar) (*n* = 7). (**C**) Resting membrane potential (RMP) values: control, −53 mV; after treatment with eugenol, −24 mV; and after wash-out, −41 mV. (**D**) Eugenol suppressed the AP frequency in TG neurons. (**E**) All data were normalized to controls (black bar) (*n* = 6). * *p* < 0.05 versus control, ** *p* < 0.01 versus control. APs: action potentials, RMPs: Resting membrane potentials.

We investigated the effects of eugenol on neuronal excitability in small-sized TG neurons. Under the condition of a whole-cell current-clamp, eugenol inhibited the generation of single AP following 180 pA of current injection for 5 ms; its effects were reversible after wash-out (Figure 2A,B). The resting membrane potential (RMP) measured in small-sized TG neurons was depolarized to −24 mV after eugenol treatment under the control condition (−53 mV). The effect of eugenol on RMP was relatively reversible after wash-out (Figure 2C). Additionally, we confirmed that eugenol completely suppressed the AP frequency after 90 pA for 1 s. These effects were also recovered by approximately 70% of the control level upon wash-out (Figure 2D,E).

### 3.3. Eugenol Activated Inward Current in Small-Sized TG Neurons

**Figure 3:**
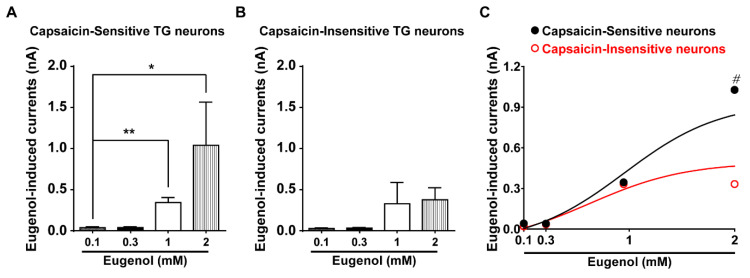
Eugenol induced inward currents in TG neurons. (**A**) Eugenol induced inward currents in capsaicin-sensitive small-sized TG neurons (15–25 μm diameter) in a dose-dependent manner (from 0.1 to 2 mM). (**B**) Eugenol induced inward currents in capsaicin-insensitive TG neurons (from 0.1 to 2 mM). (**C**) Summary of the effect of eugenol on capsaicin-sensitive and -insensitive small-sized TG neurons (*n* = 39 and *n* = 22, respectively). * *p* < 0.05 versus control, ** *p* < 0.01 versus control and # *p* < 0.05, capsaicin-sensitive versus capsaicin-insensitive.

We then performed whole-cell voltage-clamp recording with cultured small-sized (15–25 μm diameter) TG neurons that mainly express TRPV1. The eugenol-induced increase of the inward current was confirmed in a dose-dependent manner in capsaicin-sensitive TG neurons (Figure 3A). However, regardless of the dose-dependent concentration of eugenol, small inward currents were found in TG neurons with no response to capsaicin (Figure 3B). Thus, 0.1–1 mM of eugenol effected a similar inward current when these two results were compared, whereas 2 mM eugenol led to a greater inward current in capsaicin-sensitive than in capsaicin-insensitive TG neuron (Figure 3C).

### 3.4. Co-Administration of Eugenol and QX-314 Blocked VGSC Currents in Small-Sized TG Neurons

**Figure 4:**
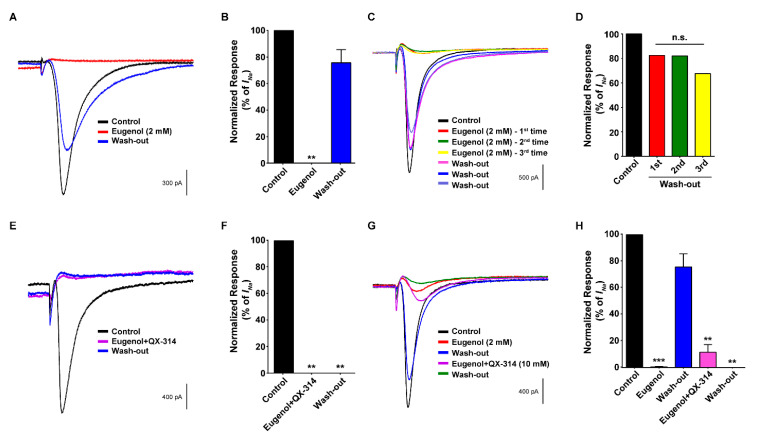
Effect of co-administration of QX-314 and eugenol on voltage-gated sodium channel currents in small-sized TG neurons. (**A**) Eugenol alone reduced voltage-gated sodium channel (VGSC) currents, which recovered after wash-out. VGSC currents were measured during 80 ms voltage steps delivered every 3 s from a holding potential of −60 mV to a test potential of 0 mV. (**B**) The proportion of VGSC current inhibition as a result of eugenol (2 mM, *n* = 15). (**C**) The interval (wash-out) time between repeated eugenol treatments (3 times) is at least 5 min. Eugenol (2 mM) inhibited VGSC currents repeatedly. After wash-out, the currents recovered to at least 70% of the control level. (**D**) The rate of VGSC current recovery after wash-out in TG neurons. The VGSC currents recovered to at least 70% of the control trace. (**E**) Representative traces of VGSC currents after the co-administration of eugenol and QX-314. QX-314 (10 mM) and eugenol (2 mM) inhibited VGSC currents, which did not recover for at least 40 min after wash-out. (**F**) The proportion of VGSC currents inhibited by eugenol and QX-314 (*n* = 8). (**G**) Eugenol alone and in combination with QX-314 suppressed VGSC currents. Trace showing the effects of eugenol (2 mM; red trace), the combination of eugenol and QX-314 (pink trace), and the control (shown as a black trace) on VGSCs. (**H**) The proportion of VGSC currents inhibited by eugenol and its combination with QX-314. ** *p* < 0.01 versus control, *** *p* < 0.001 versus control and n.s: not significant.

As shown in Figure 4A,B, eugenol completely suppressed the VGSC current, but the eugenol-induced inhibitory effect immediately disappeared after the wash-out. To further confirm these results, eugenol treatment and wash-out were performed thrice in the same TG neurons. The inhibition of VGSC currents after repeated eugenol application was reversed after the wash-out (Figure 4C,D), implying a short-lasting blockade by eugenol in TG neurons. We subsequently treated the TG neurons simultaneously with eugenol as an agonist of TRPV1 and QX-314, which can enter cells through the pores of activated TRPV1. The co-administration of eugenol and QX-314 dramatically inhibited VGSC currents, and the blockade of VGSC currents was maintained throughout the recordings up to at least 40 min after the wash-out (Figure 4E,F). In addition, we compared the effects of treatment with eugenol alone and wash-out followed by the co-administration of eugenol and QX-314 and wash-out in the same TG neuron on VGSC. As a result, eugenol alone inhibited VGSC currents, which recovered to a level similar to the control level through wash-out. The subsequent co-administration of eugenol and QX-314 completely blocked the recovery of VGSC currents even after the wash-out (Figure 4G,H).

### 3.5. Co-Administration of Eugenol and QX-314 Blocked Aps in Small-Sized TG Neurons

**Figure 5:**
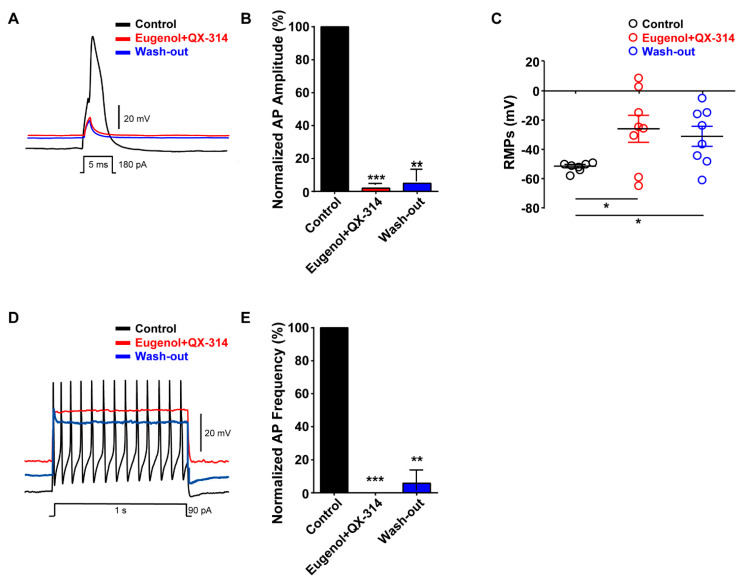
Effect of the co-administration of QX-314 and eugenol on APs in small-sized TG neurons. (**A**) Combination of eugenol (2 mM) and QX-314 (10 mM) completely abolished the AP generation even with current injection (180 pA, 5 ms). (**B**) Average percentage amplitudes of single APs after co-administration of eugenol and QX-314. (**C**) Average resting membrane potentials in the control, −53 mV; after co-administration of eugenol and QX-314, −26 mV; and after wash-out, −31 mV. (**D**) Eugenol and QX-314 reduced the AP frequency, which remained blocked even after wash-out. (**E**) All data were normalized to control (black bar). Data are presented as mean ± standard error of the mean (*n* = 8). Statistically significant differences from co-administration of eugenol and QX-314 or after wash-out in B and C. * *p* < 0.05 versus control, ** *p* < 0.01 versus control, *** *p* < 0.001 versus control.

We finally examined the effect of the co-administration of eugenol and QX-314 on neuronal excitability in small-sized TG neurons. Their co-administration almost abolished the generation of the single AP, and the inhibitory effect was maintained after wash-out (Figure 5A,B). The RMP was depolarized to −20 mV in the presence of eugenol and QX-314; it did not return to the control level after the wash-out (Figure 5C). Additionally, the co-administration of eugenol and QX-314 completely suppressed the AP frequency, and the inhibitory effect also remained after wash-out (Figure 5D,E). Therefore, we demonstrated that a prolonged inhibition of VGSC current was induced by the co-administration of eugenol and QX-314 in TG neurons. These results showed that the combination of eugenol and QX-314 achieved a more prolonged and irreversible effect than did eugenol alone in small-sized TG neurons.

## 4. Discussion

TG neurons express a variety of ion channels and receptors involved in the pain detection, propagation and transmission [TRP channels, purinergic P2X receptors, acid sensing ion channels (ASICs), PIEZOs, VGSCs, and voltage gated calcium channels (VGCCs), etc] [22,23,24,25,26]. TRP channels, including TRPV1, transient receptor potential ankyrin 1 (TRPA1), and transient receptor potential melastatin 8 (TRPM8), are expressed in nociceptive neurons, where they act as specific receptors for multiple and distinct nociceptive stimuli [27,28,29]. VGSCs regulate the generation and propagation of AP in sensory neurons such as TG [30,31,32]. Small-sized TG neurons are composed of unmyelinated C-fibers that express VGSCs (e.g., Nav1.7 and Nav1.8); the main ion channels responsible for pain sensation in the trigeminal system [13,21,30,32]. Therefore, controlling the excitability of nociceptive TG neurons by modulating VGSCs is potentially useful for the management of physiological or pathological pain in the orofacial area. For this reason, eugenol and QX-314 are studied as LA and VGSC blockers, respectively, to effect pain control.

Eugenol features a variety of analgesic and anti-inflammatory properties that render its application useful in dental practice [12,13,15,33]. However, Eugenol’s mechanism of action in different sizes of target TG neurons has not yet been studied in detail. In the peripheral system, eugenol has been used as an analgesic drug because it can inhibit VGSC currents [15], high-voltage-activated calcium channel currents (HVACC) [34,35], voltage-gated potassium channel currents [36], hyperpolarization-activated cyclic nucleotide-gated channels (HCN) [20], and ATP-induced P2X(3) currents [14] in the dental primary afferent neurons. In sensory neurons, there are two general classes of VGSC: TTX-sensitive (TTX-s INa) and TTX-resistant (TTX-r INa) [13,31,32,37]. Most of the TTX-s INa are exhibited on small-sized TG neurons, while TTX-r INa primarily on large-sized TG neurons [38,39]. Thus, eugenol may extensively inhibit both types of VGSC expressed in the sensory neurons of various sizes. Additionally, among the calcium channel subtypes of sensory neurons, eugenol preferentially blocks the N-type calcium channel, the major subtype of HVACC [34,40]. Interestingly, the inhibitory range of eugenol on HVACC (~10^−4^ to 5 × 10^−3^ M) corresponds to that used in dental therapeutic applications [34]. The concentration of eugenol that is required to inhibit HVACC is higher than that of INa inhibition [12,15,39]. Therefore, the voltage-gated sodium channels are more sensitive to eugenol than HVACC in sensory neurons. The P2X subtypes play an important role in pain signaling in sensory neurons [14,41]. P2X3, the main P2X subtype of TG neurons, is primarily localized to small-sized TG neurons. Eugenol inhibits P2X3 current in the trigeminal ganglion neurons [14]. The identification of a hyperpolarization-activated current (Ih) in medium and large-sized TG and dorsal root ganglion (DRG) neurons and a relatively weak amplitude of current (Ih) in small-sized neurons suggests that the HCN channel may also play an important role in mechanical allodynia of neuropathic pain [20]. Therefore, eugenol probably inhibits VGSC (TTX-s INa), HVACC, and P2X3, but not HCN, in small TG neurons; the pain regulating mechanism of eugenol used in dentistry may be attributable to its inhibition of these channels.

**Figure 6:**
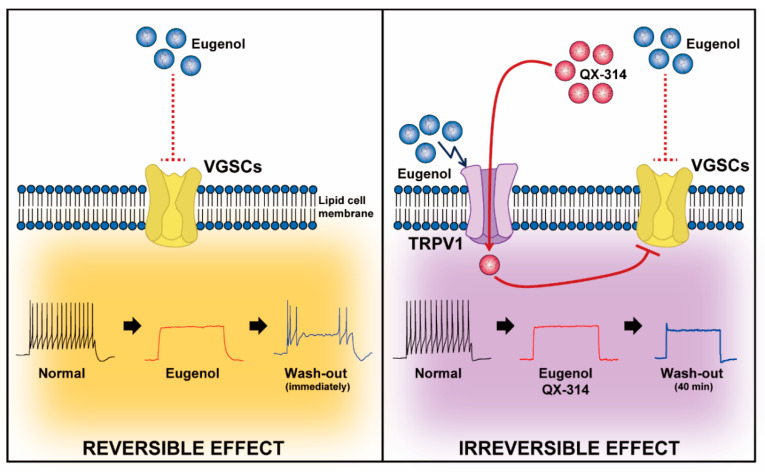
Comparison of reversibility between eugenol alone and co-application of eugenol and QX-314. The neuronal VGSCs and TRPV1 channels, which are the primary pain-sensing elements in pain sensation, were both expressed in sensory TG neurons. Reversible effect: The inhibition of VGSCs and AP by eugenol alone were relatively reversible after wash-out. Irreversible effect: Eugenol stimulated TRPV1 channels, then QX-314 could enter into the TG neurons via TRPV1 channels. The QX-314 suppressed the activity of VGSCs and AP, but the inhibitory effect irreversibly maintained even after wash-out.

The TRPV1 channel is a non-specific cation channel expressed in the peripheral and central nervous system [42,43]. It can be activated by various exogenous and endogenous stimuli such as capsaicin, hot temperature, low pH, inflammatory mediators, arachidonic acid metabolites, ATP, anandamide, and lipoxygenase products [7,8,9,10,11,12]. Thus, TRPV1 plays an important role in pain transduction under physiological and pathological conditions [44,45]. The combined use of QX-314 and capsaicin is the product of a trend towards identifying safe and effective LAs capable of selectively blocking activity from nociceptive afferent fibers [1,13,16,46,47]. The extraneuronally applied combination of capsaicin with low concentration of QX-314 opens the TRPV1 channel, which then undergoes pore dilation upon activation and allows the permeation of large cations, such as gentamicin and QX-314 [48,49]. Thus, QX-314 can enter cells through the TRPV1 receptors in the capsaicin-responsive DRG neurons and selectively inhibit VGSCs [50,51,52]. Similar findings were also reported in TRPV1-expressing TG neurons; capsaicin and QX-314 blocked the sodium current and Aps, and these actions were irreversible even after washing [1,10,53]. The potential advantages of this approach include the selective anesthesia of capsaicin-sensitive (TRPV1 expression) sensory neurons. Various LAs, such as lidocaine, bupivacaine, acidic solution, and surfactants, are also combined with QX-314 as TRPV1 activators [11]. However, beyond effecting greater analgesia, improvements in LAs are required to decrease toxicity [6,54,55]. In addition, the use of LAs is limited by the duration of its action and its dose-dependent adverse effects [56,57,58,59]. LAs are often used in combination with other Las, owing to the synergistic effect of their combination in prolonging the duration of action, separating the sensory-motor block, or limiting the cumulative dose requirement of LAs [60]. Although eugenol could be an effective LA in the field of dentistry [12,13,61], it has a short duration of action and non-selectively blocks various channels (Na and calcium) in TG neurons of various sizes [62]. Therefore, eugenol alone is ineffective in the management of chronic or persistent pain [12,35,62,63]. We confirmed the possibility of using a combination of eugenol and QX-314 in TG neurons to induce a stronger and longer analgesic effect. The present results are similar to those reported by a study in which the co-administration of eugenol and QX-314 induced a more persistent analgesic effect in TG neurons than did eugenol alone [10]. The inhibitory effects of eugenol on VGSC currents and APs disappeared after the wash-out, supporting the notion of short-lasting sensory neuron block [12,15,39]. However, eugenol administered in combination with QX-314 induced a sustained inhibitory effect even after a long wash-out period, producing irreversible analgesic effects in the trigeminal system (Figure 6).

Since eugenol activates other channels such as TRPA1 as well as TRPV1 [12,17,39,64], QX-314 can enter through eugenol-activated channels [1]. Eugenol (1 mM) induced increases in the level of intracellular calcium by the activating TRPA1 regardless of the expression of TRPV1 in TG neurons [64]. Our results showed that eugenol (2 mM) significantly elicits larger inward currents in capsaicin-sensitive TG neurons than capsaicin-insensitive TG neurons (Figure 3). This implies that 2 mM eugenol produces an inward current through TRPV1 activation in capsaicin-sensitive neurons. In addition, we confirmed the percentage of each physiological function of the various channels was TRPV1 (75%), TRPA1 (20%), TRPM8 (30%), TRPV1/TRPA1 (20%), and TRPV1/TRPM8 (15%) in small-sized TG neurons (Dil-labeled) [65]. Thus, the low expression of TRPA1 is probably insufficient to promote the entry of QX-314 in small TG neurons. A study of the transportability of QX-314 to sensory neurons via TRPV1 (capsaicin/QX-314), TRPA1 (AITC/QX-314), and TRPM8 (menthol/QX-314) reported that TRPA1 and TRPM8 escalated the transport of QX-314 compared to TRPV1 [66]. Although other functional mechanisms may be affected, the application of eugenol (2 mM) and QX-314 produces a specific analgesic effect on TRPV1 expressed by small sized TG neurons. As shown in Figure 1, eugenol completely inhibited the VGSC current in capsaicin-insensitive TG neurons, but the inhibitory effect disappeared upon wash-out. Eugenol has been reported to feature a variety of functions, including anti-inflammatory, antinociceptive, antioxidant, and antiapoptotic activities [61,67,68,69]. When inflamed or nerve-damaged tissues cause the dysregulation of VGCC associated with increased pain sensation in pain-inducing conditions, eugenol promotes functional recovery, alleviates pathological symptoms, and further blocks pain sensation via the inhibition of VGCC. While, eugenol is considered safe, its potential toxicity has engendered controversy [67,70]. However, nano-eugenol, delivered using nanoparticles, was demonstrated to alleviate the symptoms of inflammation with low toxicity in patients with rheumatoid arthritis [67]. In this way, studies to improve various functions of eugenol are in progress. Further research is being performed to elucidate the duration of the clinical effects of eugenol and thus improve our understanding of the most appropriate methods for using eugenol and whether it features any synergistic effects that may help to attenuate long-term pain, such as chronic post-surgical pain [71]. We have shown the promise of such applications through our possible experimental results concerning the combined use of eugenol and QX-314. Further studies are needed to elucidate such long-term effects mediated by the physiological and pathological functions of eugenol (with QX-314) in the orofacial region.

## 5. Conclusions

This study demonstrated that the co-administration of eugenol and QX-314 could exert a more prolonged and selective analgesic effect on the trigeminal system by blocking APs through VGSC inhibition. Hence, eugenol and QX-314 are potentially useful as pain-selective LAs in the orofacial region.
